# Supplementary material for: Genes with 5′ terminal oligopyrimidine tracts preferentially escape global suppression of translation by the SARS-CoV-2 Nsp1 protein
Source: RNA. 2021 Sep;27(9):1025–45. doi: 10.1261/rna.078661.120 (PMC8370740; doi:10.1261/rna.078661.120)
Supplement: Supplemental Material [file supp_27_9_1025__DC1.html]

Genes with 5′ terminal oligopyrimidine tracts preferentially escape global suppression of translation by the SARS-CoV-2 Nsp1 protein — Supplemental Material 

# Genes with 5′ terminal oligopyrimidine tracts preferentially escape global suppression of translation by the SARS-CoV-2 Nsp1 protein

## Supplemental Material

- Supplemental\_Figures.pdf
- Supplemental\_Files.xlsx
- Supplemental\_Information.docx
