# Supplementary material for: Genes with 5′ terminal oligopyrimidine tracts preferentially escape global suppression of translation by the SARS-CoV-2 Nsp1 protein
Source: RNA. 2021 Sep;27(9):1025–45. doi: 10.1261/rna.078661.120 (PMC8370740; doi:10.1261/rna.078661.120)
Supplement: Supplemental Material [file supp_078661.120_Supplemental_Figures.pdf]

Supplemental information

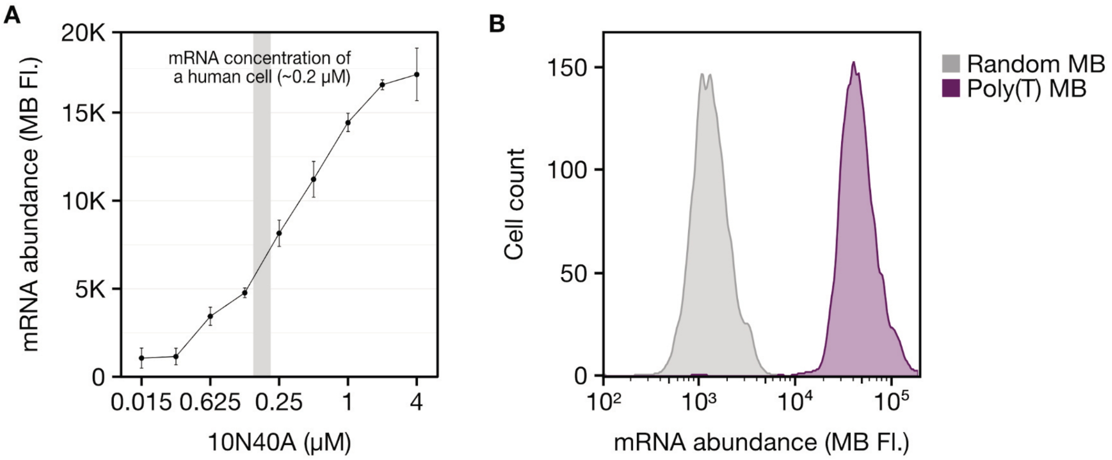

Supplemental Figure 1 - Validation of sensitivity and specificity of the molecular beacon

(A) *In vitro* molecular beacon (MB) assay targeting the 10N40A synthetic oligo. The gray region indicates the estimated concentration of mRNA in a cell. **(B)** Signal to noise ratio of the MB signal using a random (gray) and specific MB (purple) with HEK293T cells.

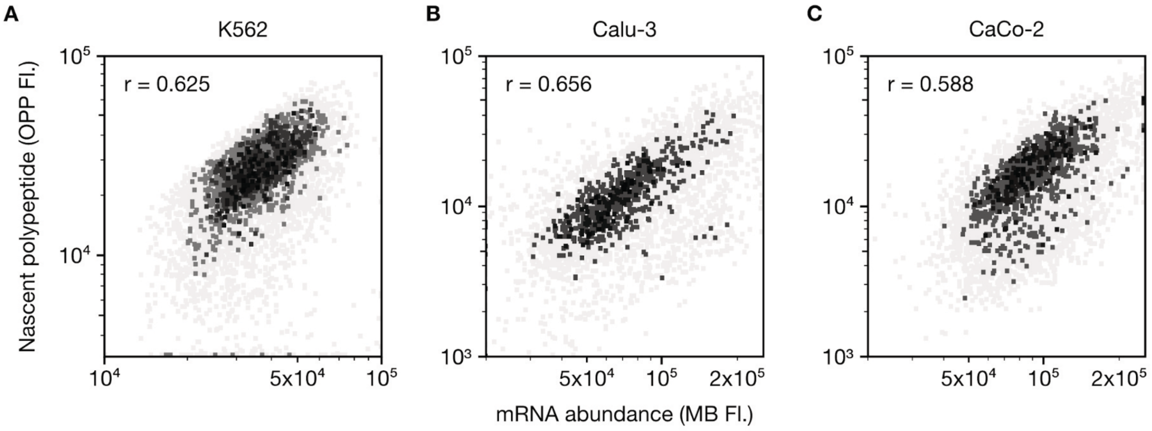

Supplemental Figure 2 - MeTaFlow assay of different cell lines

(A) MeTAflow assay of K562 cell line, **(B)** Calu-3 cell line, and **(C)** CaCo-2 cell line after OPP (50 µM) treatment for 10 minutes (K562 cell line) or for 30 minutes (Calu-3 & CaCo-2 cell line).

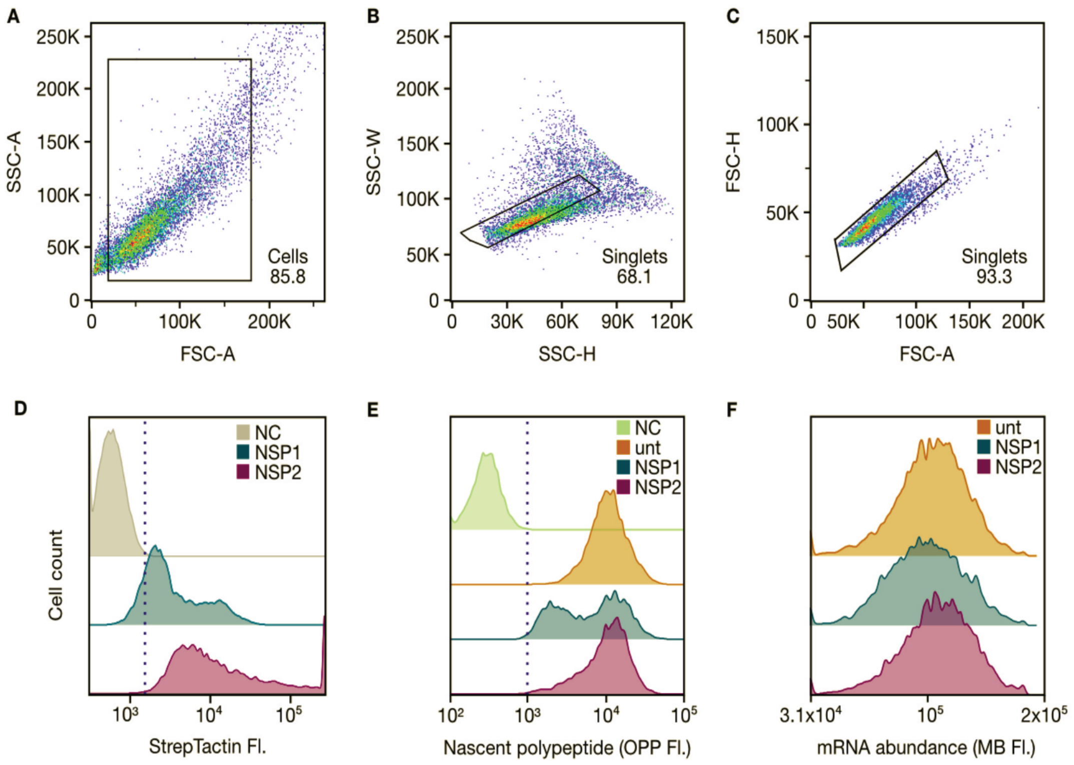

Supplemental Figure 3 - Gating strategy for MeTAFlow assay

(A) FSC-A & SSC-A gating followed by doublet discrimination using the **(B)** SSC-H & SSC-W plots and **(C)** FSC-A & FSC-H plots **(D)** The DY-549 signal in Nsp1 and Nsp2-transfected cells during the MeTAFlow assay. The DY-549 positive population in the Nsp1 and Nsp2 conditions were gated (dotted line) based on the negative control StrepTactin-DY549 background signal (NC, gray). **(E)** AF488 signal in untransfected control (unt), Nsp1 and Nsp2 conditions during the MeTAFlow assay. The OPP-AF488+ population in Nsp1, Nsp2 and untransfected control conditions were gated (dotted line) based on the negative control AF488 background signal (NC, green). **(F)** The Cy5 molecular beacon signal in control, Nsp1-, and Nsp2-transfected HEK293T cells during the MeTAFlow assay. Compensation was carried out using single stained

HEK293T controls. OPP-treated cells, Nsp2-transfected cells, and cells incubated with MB were used as AF488, DY549, and Cy5 single stained controls, respectively.

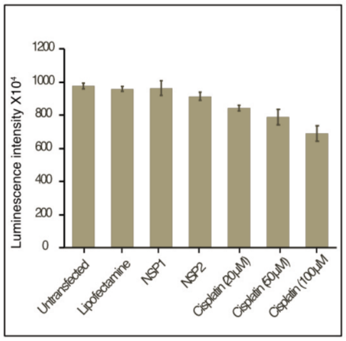

Supplementary Figure 4 - Viability of cells expressing Nsp1: The bar plot represents mean luminescence intensity ± SEM from three independent replicates. Luminescence is a reporter of viability of HEK293T cells either untransfected or transfected with Nsp1 or Nsp2 or with lipofectamine alone. Cisplatin treatments at different concentrations were used as positive controls for the viability assay.

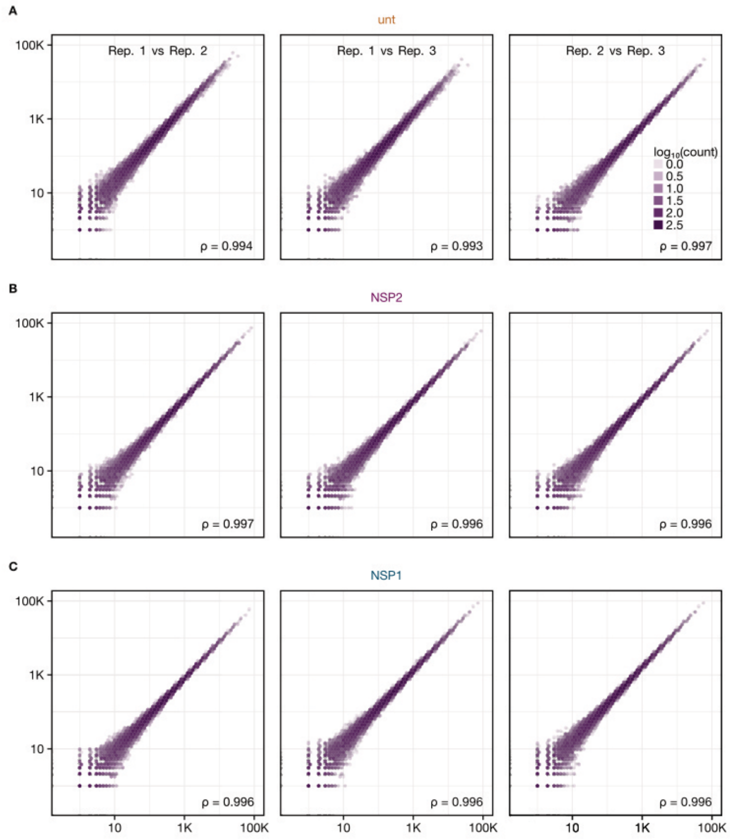

Supplemental Figure 5 - RNA-Seq correlation plots

(A) Pairwise comparison of gene expression data generated with untransfected (unt), **(B)** Nsp2-expressing, and **(C)** Nsp1-expressing cells. The Spearman correlation coefficients (ρ) are provided and the density of transcripts is represented with the color scale. The axes correspond to the number of reads mapping to the CDS region of a gene for the replicates being compared.

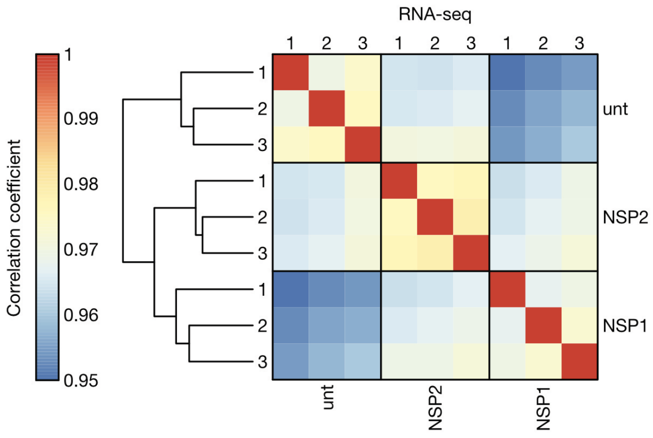

Supplemental Figure 6 - Clustering of RNA-Seq correlations across replicates

Hierarchical clustering of the pairwise Spearman correlation values between RNA-Seq replicates 1, 2, and 3 of untransfected (unt), Nsp2-transfected (Nsp2), and Nsp1-transfected (Nsp1) conditions. RNA-Seq read counts mapping to the coding region of each analyzed transcript were used to calculate pairwise Spearman correlation coefficients.

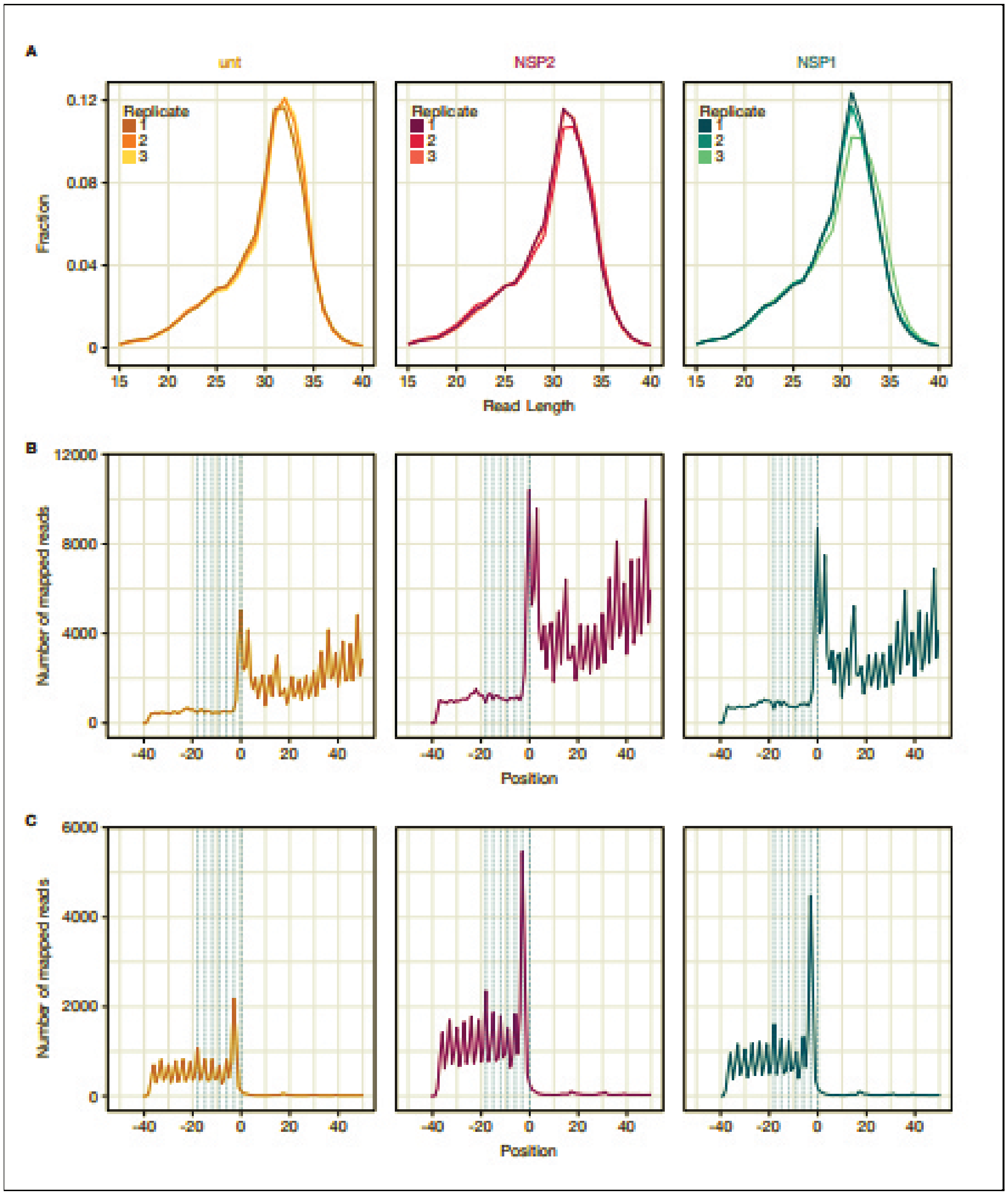

Supplemental Figure 7 - Quality control metrics for ribosome profiling data from HEK293T cell line

A) Read length distributions. Ribosome profiling read lengths for untransfected (unt), NSP2, and NSP1 conditions were quantified as fractions of the total reads. All experiments were performed in triplicate. Only reads which mapped to the CDS region of the genes were used. (B) Representative metagene plots of the translation start site across conditions, each from the first

replicate. Position 0 denotes the start site and is flanked by a 40 nt region downstream and 50 nt region upstream. Mapped reads, with matching positions relative to the translation start site, were aggregated. Positions of mapped reads were adjusted according to their p-site offsets. The x-intercept of the vertical dashed lines are at positions -18, -15, -12,..., -3, 0, indicating 3-nt periodicity. (C) Metagene plots of the translation stop site across conditions. These plots were generated as in panel B, where position 0 denotes the stop site.

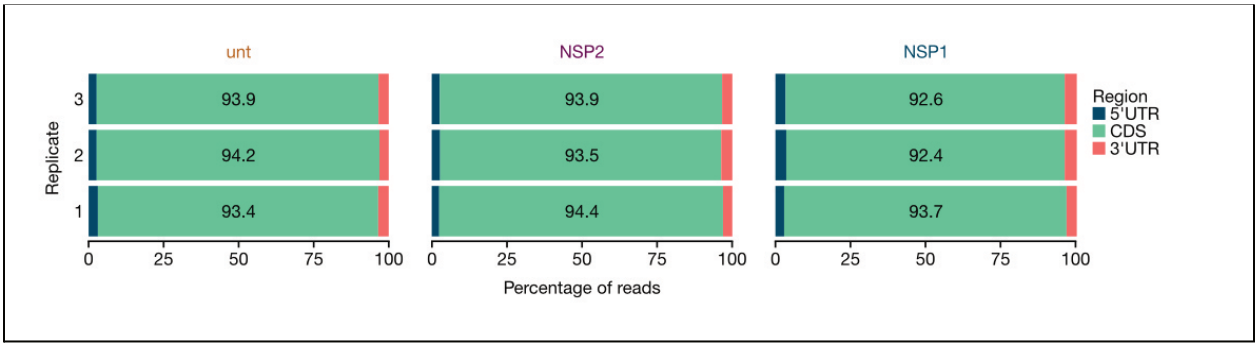

Supplemental Figure 8 - Percentages of region counts as basic QC for ribosome profiling

Representation of the percentage of ribosome profiling reads that mapped to different transcript regions: 5’ UTR, CDS, and 3’ UTR. Reads with lengths 28-35 nt were plotted for three replicates of untransfected control (unt), Nsp2, and Nsp1 conditions.

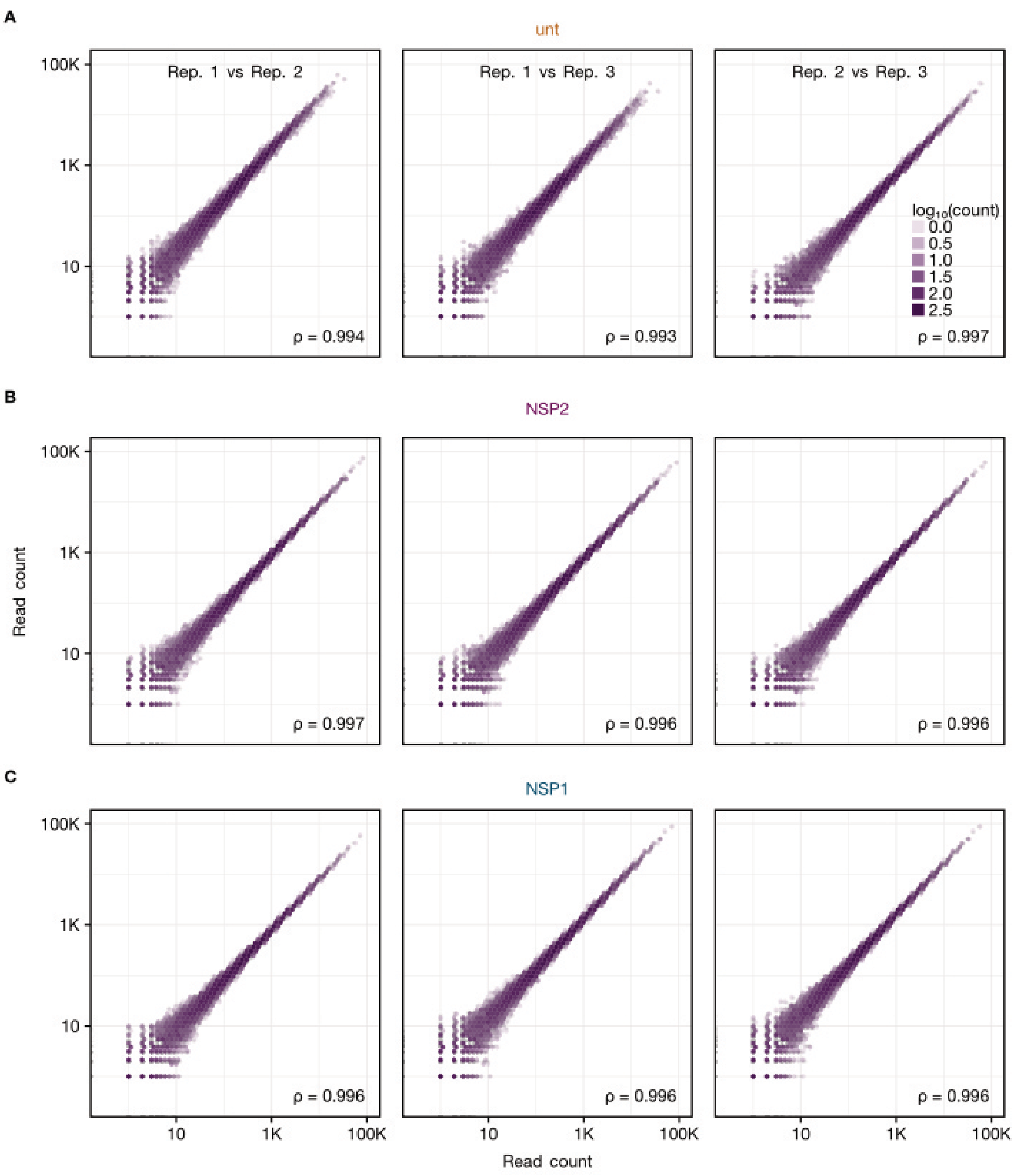

Supplemental Figure 9 - Ribosome profiling correlation plots

(A) Pairwise comparison of ribosome occupancy data generated with untransfected (unt), **(B)** Nsp2-expressing, and **(C)** Nsp1-expressing cells. The Spearman correlation coefficients (ρ) are provided and the density of transcripts is represented with the color scale. The axes correspond

to the number of ribosome footprints mapping to the CDS region of a gene for the replicates being compared.

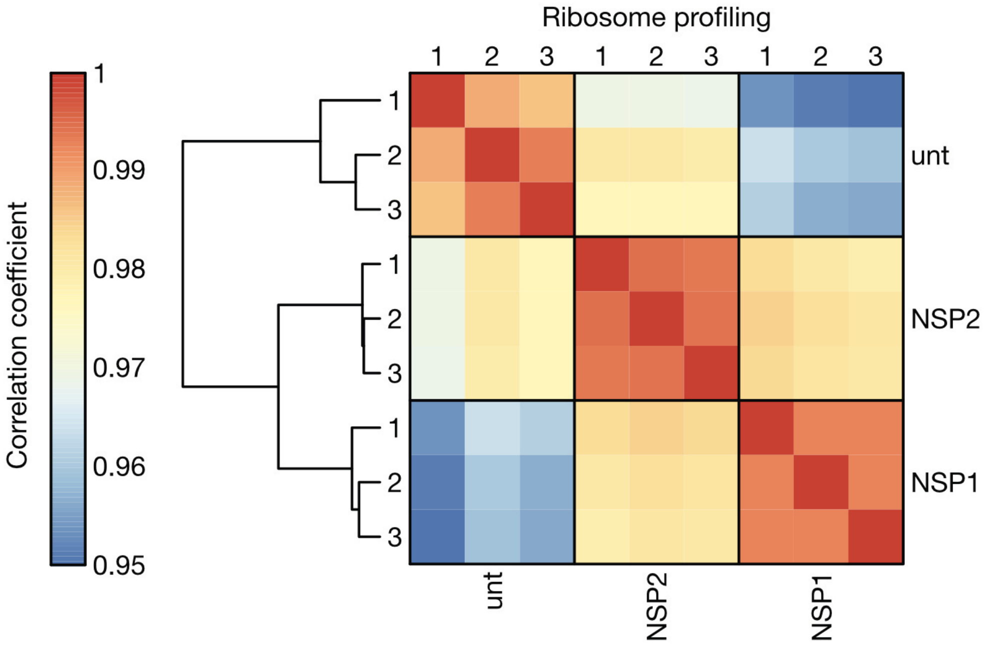

Supplemental Figure 10 - Clustering of ribosome profiling correlations across replicates from HEK293T cell lines

Hierarchical clustering of the pairwise Spearman correlation values between ribosome profiling replicates 1, 2, and 3 of untransfected (unt), Nsp2-transfected (Nsp2), and Nsp1-transfected conditions. Ribosome profiling read counts mapping to the coding region of each analyzed transcript were used to calculate pairwise Spearman correlation coefficient.

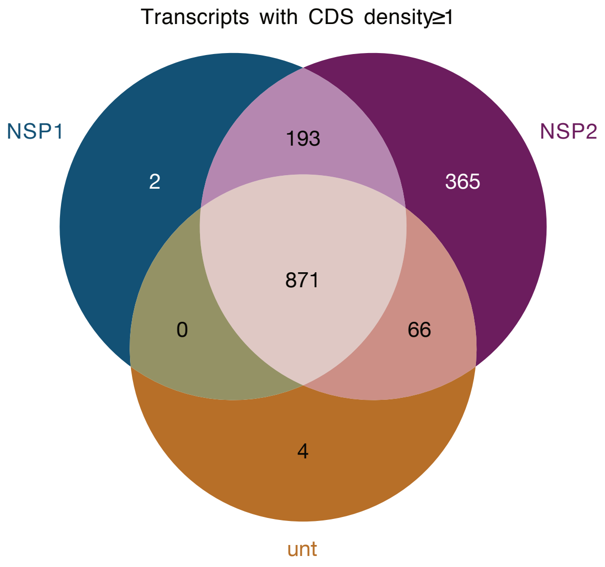

Supplemental Figure 11 - Sets of highly covered transcripts from ribosome profiling

Transcripts with CDS density ≥1 (total number of CDS mapping ribosome footprints divided by CDS length) in untransfected (unt), Nsp2, and Nsp1 conditions. For nucleotide coverage analyses, we used the union of all three sets.

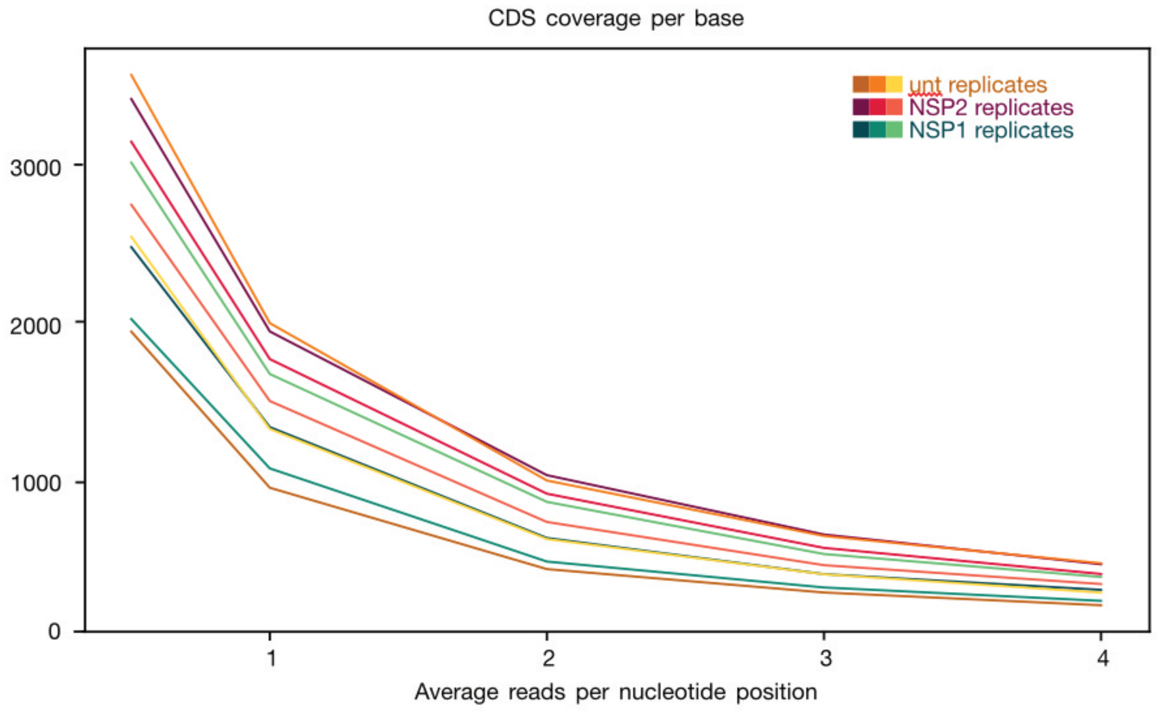

Supplemental Figure 12 - Levels of highly covered transcripts

Average CDS coverage by number of transcripts. For each transcript, the total number of footprints on the CDS was divided by the length of CDS, giving us the average number of reads per nucleotide position, shown in the x-axis. The y-axis shows the number of genes exceeding the given average reads per nucleotide. In other words, the existence of a point (x, y) means that there are y genes whose average per nucleotide is $\geq x$.

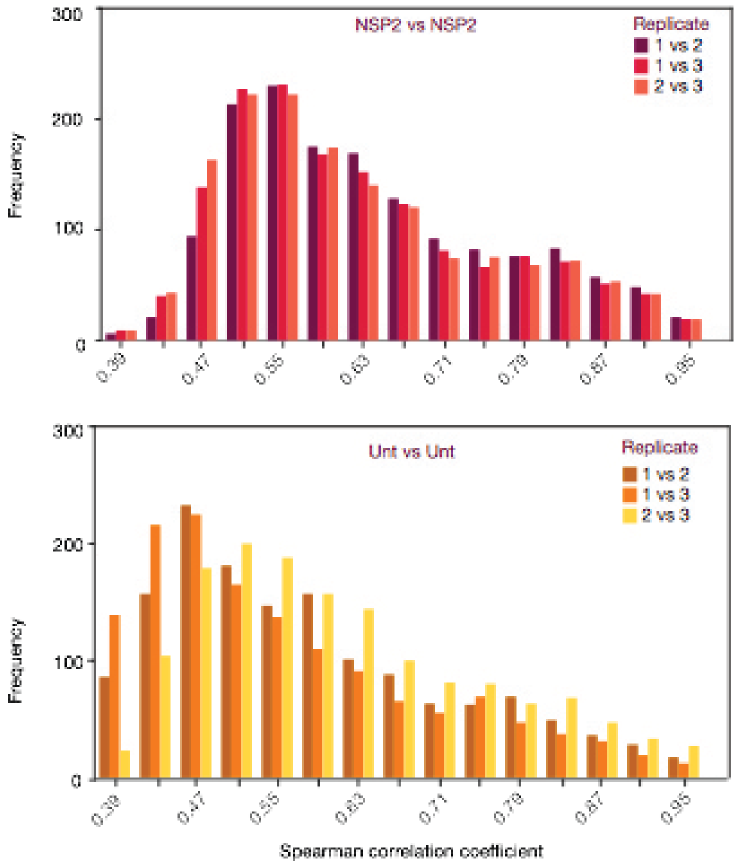

Supplemental Figure 13 - Ribosome occupancy at nucleotide resolution

(A) Histogram of the Spearman correlations for all pairwise replicate combinations of Nsp2-expressing cells. The Spearman correlation coefficient was calculated for each pair of replicates, for each gene, and plotted against the frequency of occurrence. **(B)** Histogram of the Spearman correlations between all pairwise replicate combinations of untransfected (unt) cells.

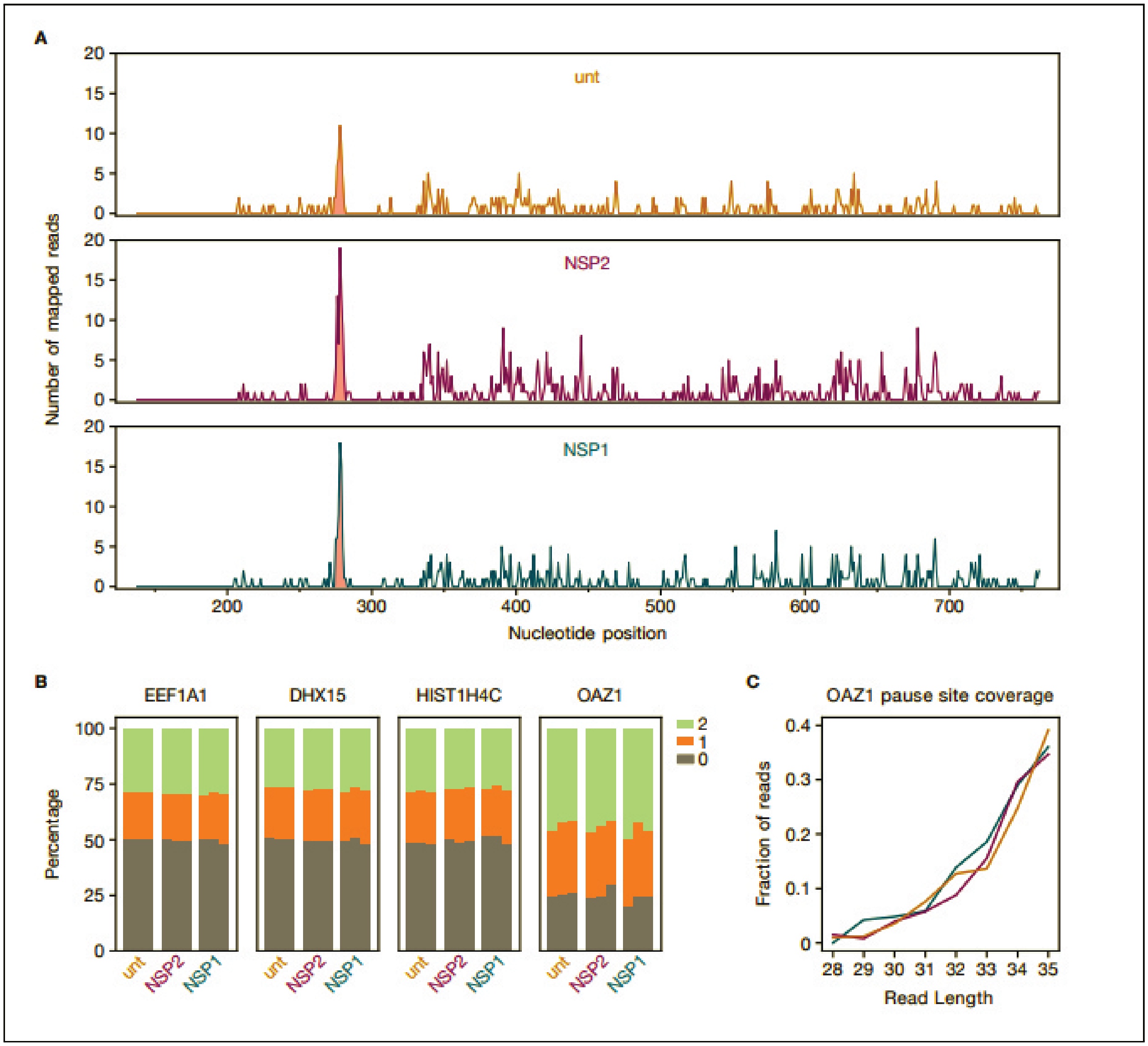

Supplemental Figure 14 - Analysis of frameshifting in the OAZ1 gene

(A) CDS Coverage of the OAZ1 gene. The x-axis shows the nucleotide position and the y-axis shows the number of ribosome footprints. The pause site, spanning the positions from 259 to 270 nt, is highlighted in red. Coverages from the first replicate of each condition are plotted individually. **(B)** Frame shift analysis. The coding sequence was partitioned into bins of 3 nucleotides (0 nt, 1 nt and 2 nt). For EEF1A1 and DHX15, the ribosome footprints were aggregated at each frame position, in the entire CDS. Then, the percentages of the frame

positions were calculated. For OAZ1, ribosome footprints to the right of the pause site shown in A were aggregated. (**C)** Coverage per read length at the pause site. For each read length from 28 to 35 nt, the number of reads at the pause site were divided by the number of all reads having the same read length, resulting in the ratio shown on the y-axis.

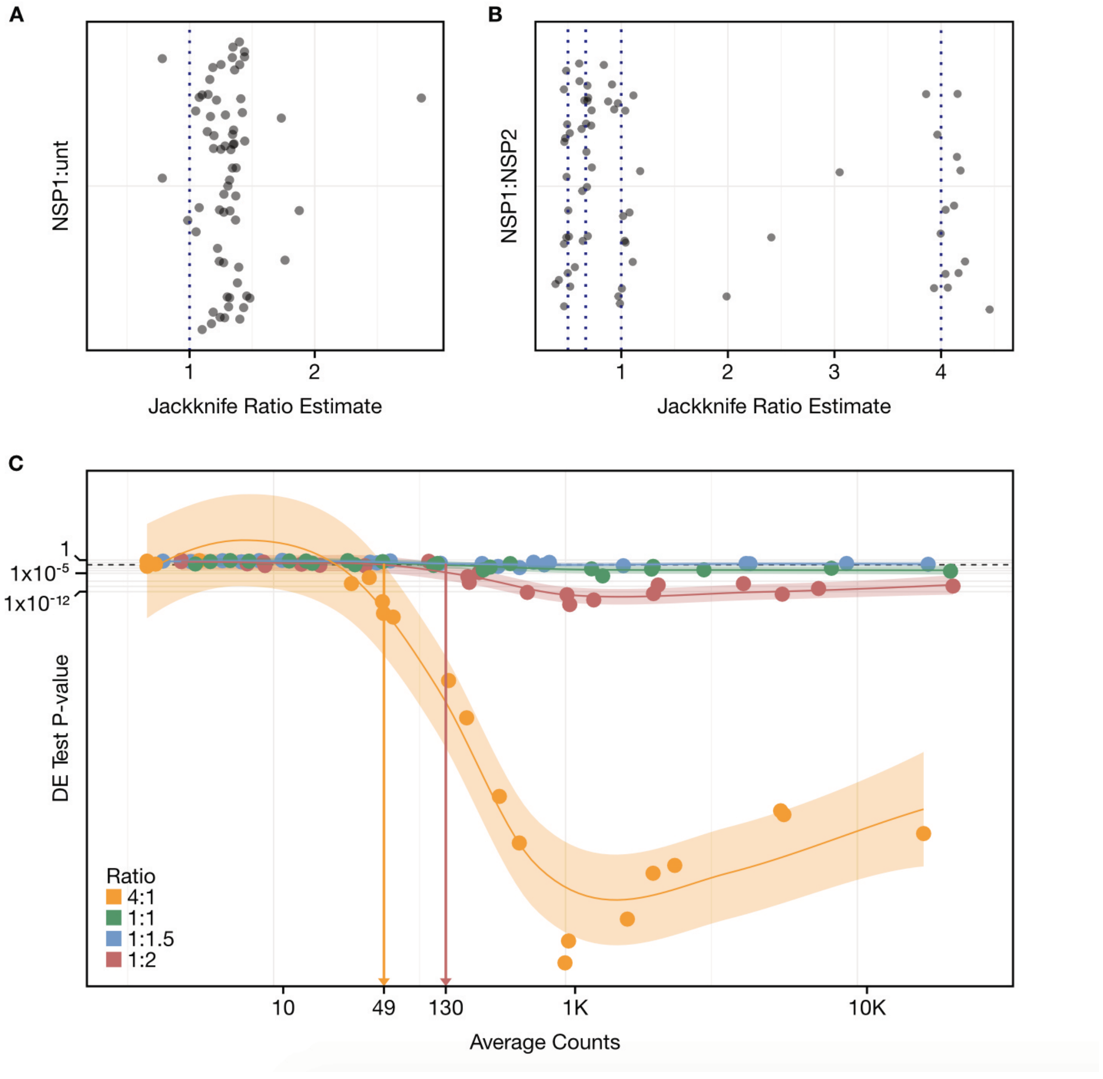

Supplemental Figure 15 - ERCC spike-in RNA controls

(A) Jackknife estimate of the observed ratio for each spike-in control was calculated for RNA sequencing libraries prepared from Nsp1-expressing (Nsp1) HEK293T cells and untransfected (unt) controls in the presence of ERCC Spike-in Control Mix 1 or **(B)** Nsp2-expressing HEK293T

cells prepared in the presence of ERCC Spike-in Control Mix 2. An equal mRNA fraction between samples would lead the ratio estimates to be centered at 1. A higher ratio indicates lower mRNA content in cells expressing Nsp1. Different spike-in controls are designed to be at four distinct ratios indicated by the dashed lines. **(C)** Differential expression p-value for each spike-in plotted against its average count. ERCCdashboard was used to estimate a limit of detection of ratios for each of the spike-in RNAs. Vertical lines indicate the threshold read count above which differential expression of the given magnitude can be confidently identified.

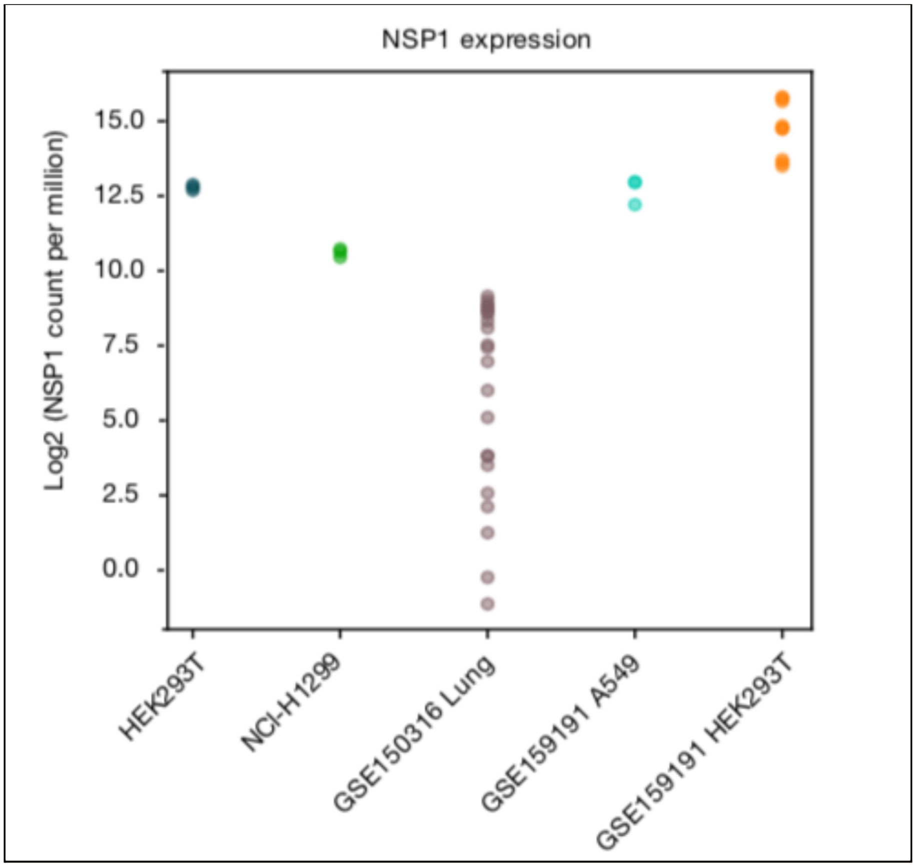

Supplemental Figure 16: Comparison of NSP1 gene expression across different cell types

RNA-Seq data from the current study (HEK293T and NCI-H1299 transfected with Nsp1) and two publicly available datasets are mapped to the NSP1 sequence and the human

transcriptome. Each data point represents an experiment from the indicated study. For each experiment, the number of reads mapping to NSP1 was divided by the total number of mapped reads in that experiment, and then multiplied by one million. GSE150316 contains RNA-Seq data from human SARS-CoV-2 infected lung tissues. (Weingarten-Gabbay et al. 2020) (GSE159191) included experiments from lung carcinoma cells (A549) and human embryonic kidney cells (HEK293T) infected with SARS-CoV-2.

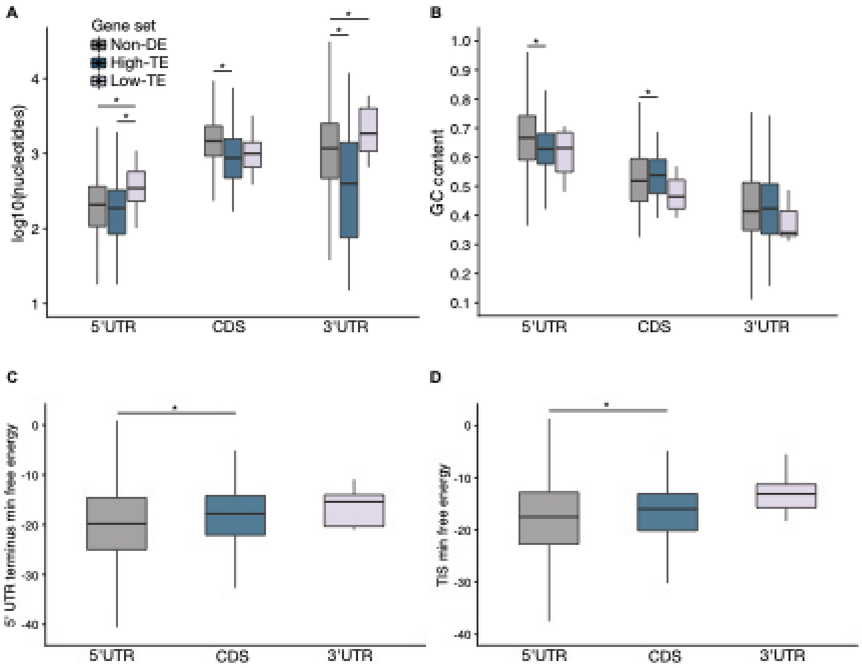

Supplemental Figure 17 - Sequence feature analysis of differentially translated genes in the presence of Nsp1

(A) 5' and 3' untranslated region (5'UTR, 3'UTR) and coding sequence (CDS) lengths for high and low translation efficiency (high-TE, low-TE) and non-differentially expressed (non-DE) control genes. **(B)** GC content. **(C)** Predicted minimum free energy (MFE) in -kcal/mol of the first 60 nts of the 5' UTR for comparative gene sets. **(D)** Predicted MFE for a window between -30 to +30 around the start codon. **(A-E)** Asterisks indicate significant Dunn's post-hoc tests at a FDR of 0.05. Outliers greater than (1.5 * interquartile range) were omitted for clarity.

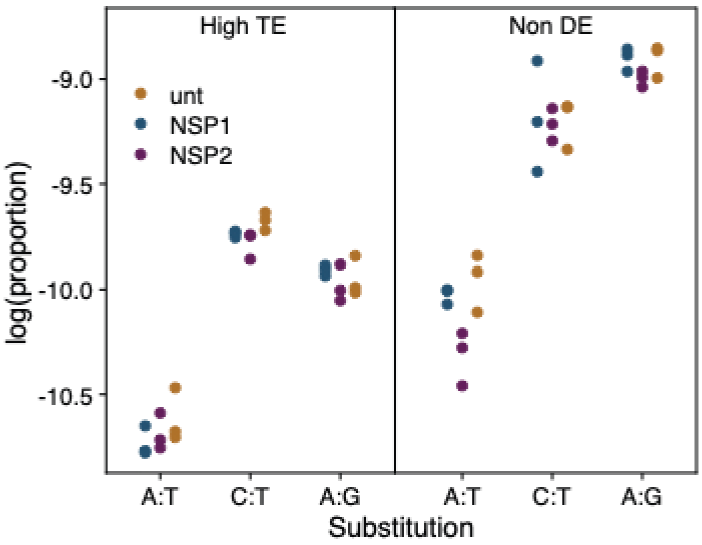

Supplemental Figure 18 - Investigation of RNA editing in high-TE genes

Base substitution frequencies in RNA-Seq alignments. Substitutions were enumerated relative to the annotated transcript strands, requiring a base quality >= 35. The counts were normalized to the total mapped read count of the library. Data was then unit-normalized by dividing by the sum of all depth-normalized matches and substitutions. The log proportions are shown for A:G, A:T (candidate ADAR editing) and C:T (candidate APOBE editing).

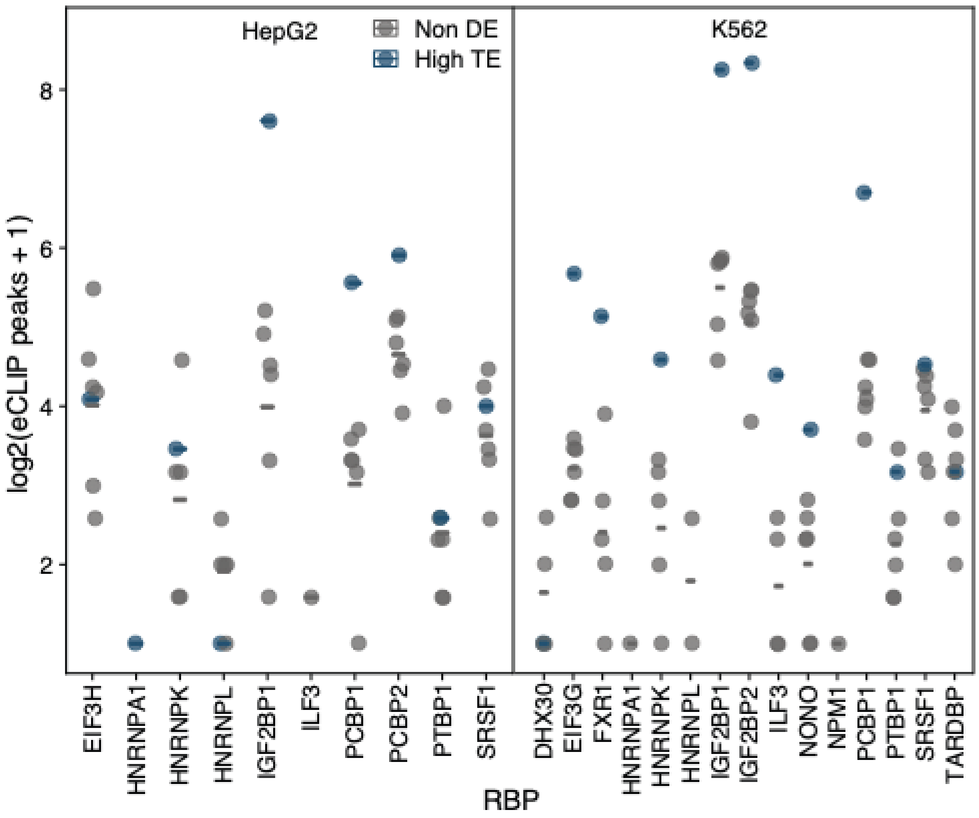

Supplemental Figure 19 - Raw data for eCLIP

Processed eCLIP data. Lines represent the median values which correspond to the heat fill in Figure 4E. Narrowpeak BED files from eCLIP experiments (Van Nostrand et al. 2020) for pertinent RNA binding proteins were intersected with exonic regions of the high-TE and six matched non-DE gene sets. Only BED features that were labeled with “IDR”, indicating reproducible peaks, were used in analysis. Peaks were summed across the gene sets and a pseudocount was added to generate each point.

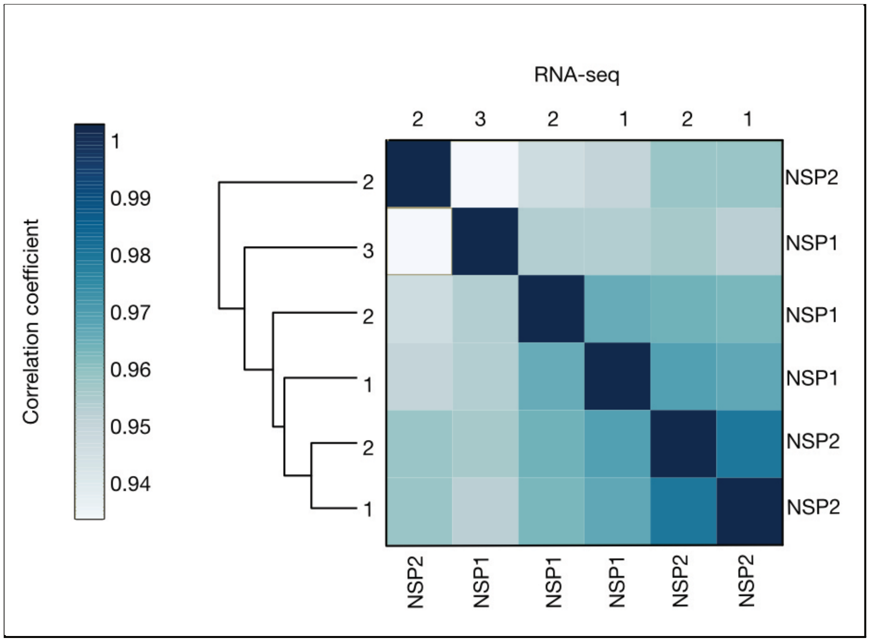

Supplemental Figure 20 - Clustering of RNA-Seq correlations across replicates in NCI-H1299 cell line

Hierarchical clustering of the pairwise Spearman correlation values between RNA-Seq replicates of Nsp2-transfected (Nsp2), and Nsp1-transfected conditions. RNA-Seq read counts mapping to the coding region of each analyzed transcript were used to calculate pairwise Spearman correlation coefficients.

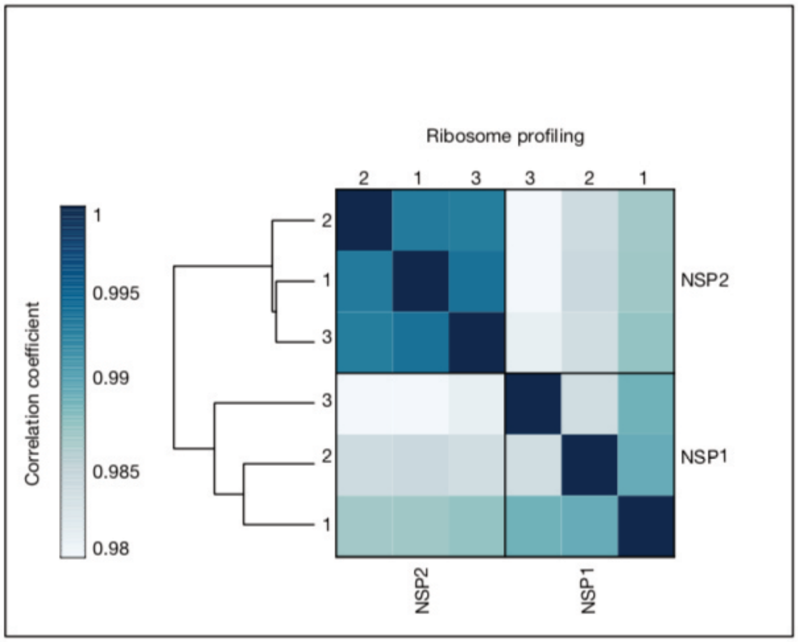

Supplemental Figure 21 - Clustering of ribosome profiling correlations across replicates in NCI-H1299 cell lines

Hierarchical clustering of the pairwise Spearman correlation values between ribosome profiling replicates of Nsp2-transfected (Nsp2) and Nsp1-transfected conditions. Ribosome profiling read counts mapping to the coding region of each analyzed transcript were used to calculate pairwise Spearman correlation coefficient.

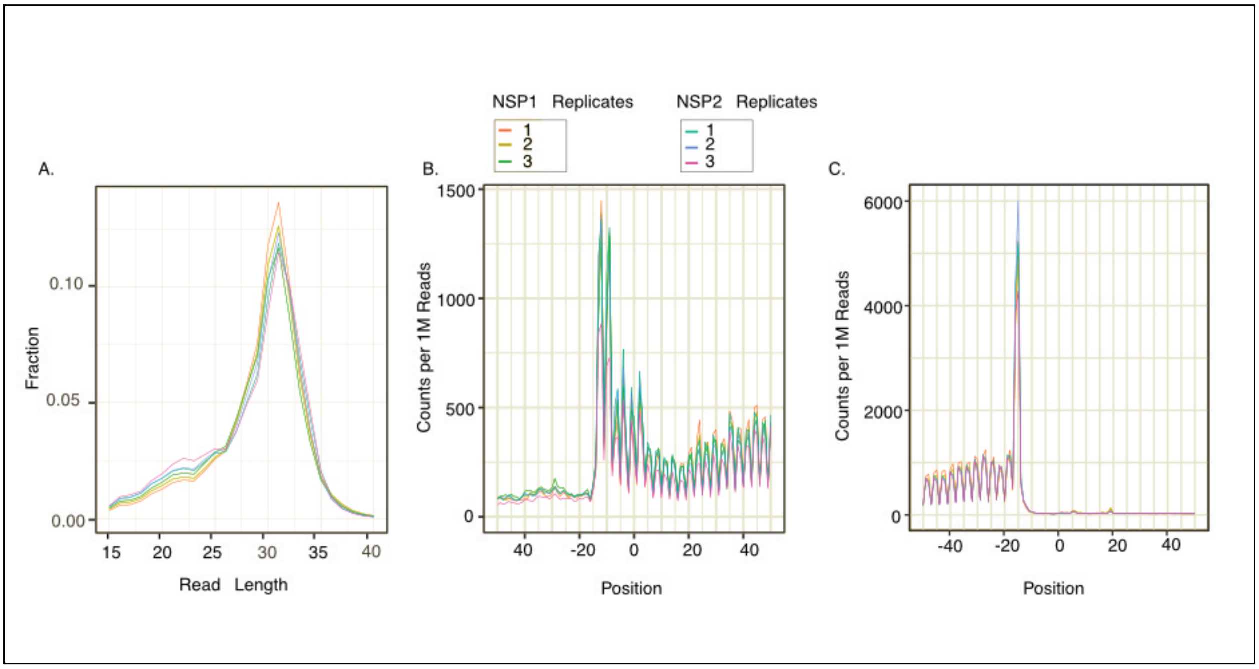

Supplemental Figure 22 - Quality control metrics for ribosome profiling data from H1299 cell line

(A) Read length distributions. Ribosome profiling read lengths for Nsp2, and Nsp1 conditions were quantified as fractions of the total reads. All experiments were performed in triplicate. Only reads which mapped to the CDS region of the genes were used. **(B)** Metagene plots of the translation start site across conditions. Position 0 denotes the start site and is flanked by a 50 nt region up and downstream. Mapped reads, with matching positions relative to the translation start site, were aggregated and normalized to counts per 1M reads (see methods). **(C)** Metagene plots of the translation stop site across conditions. These plots were generated as in panel B, but position 0 denotes the stop site. For panels B and C, the 5' end of the ribosome footprints were plotted, causing a shift of ~15 nucleotides to the left.

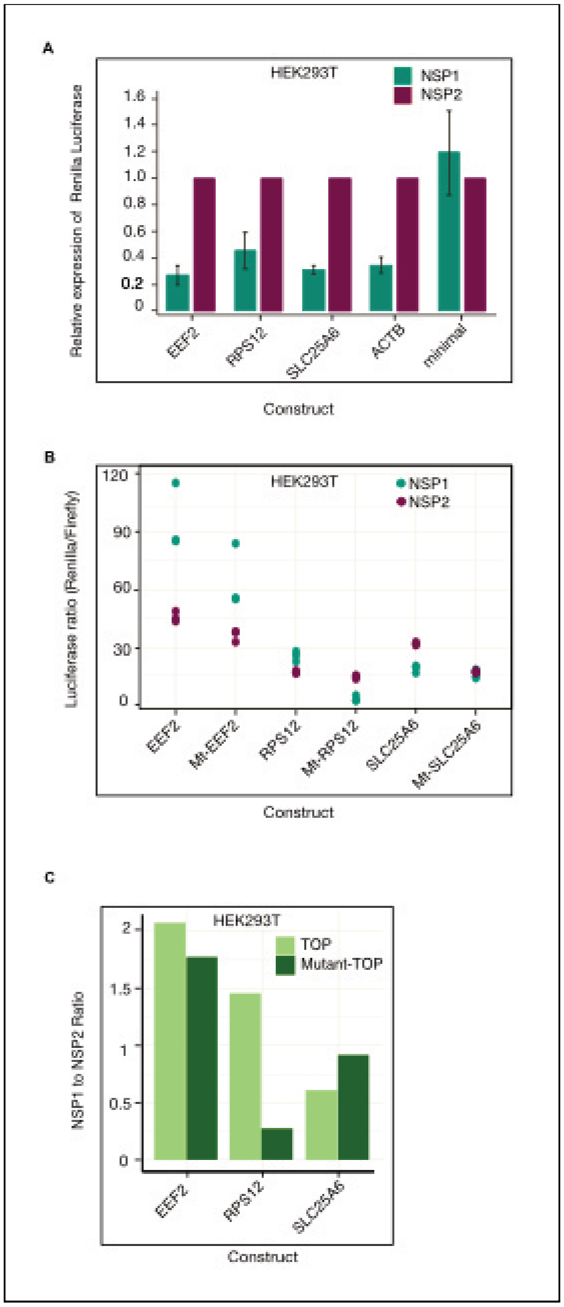

Supplementary Figure 23- Nsp1 mediated translation of TOP mRNA

(A) Relative Renilla mRNA levels from the unmodified reporter (minimal), reporter bearing UTR from non-TOP gene (ACTB) or three TOP genes (EEF2, RPS12 and SLC25A6) were analyzed by RT-qPCR in Nsp1 expressing cells and compared to the Nsp2 expressing control cells. The cotransfected Firefly luciferase plasmid was used for normalization. Bars represent the mean of

the fold change as calculated by $2^{-\Delta\Delta CT}$ and the error bars were calculated using fold changes from individual experiments (± SEM). **(B)** The ratio of Renilla luciferase to Firefly luciferase activity in HEK293T cells are plotted for three TOP constructs (EEF2, RPS12 and SLC25A6) along with their corresponding mutants (Mt) wherein the purines in the TOP motif were replaced with pyrimidines. **(C)** The mean of the ratio of luciferase values for both TOP and mutant-TOP constructs from HEK293T cells are plotted for comparison.

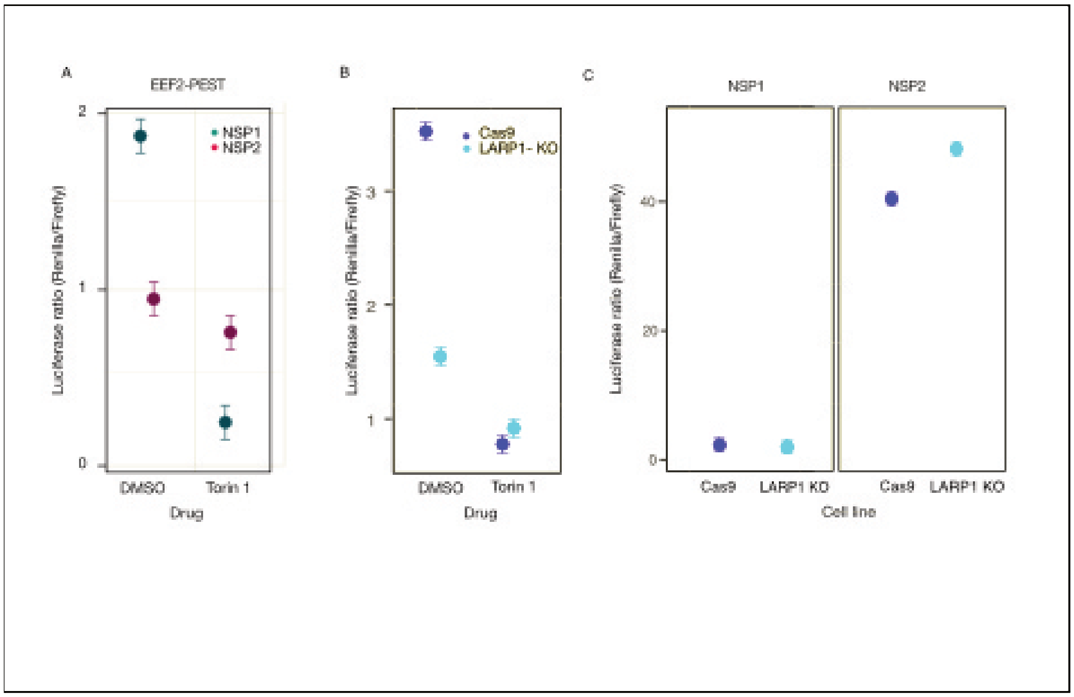

Supplemental Figure 24: Reporter assays to validate the effect of Torin 1 and LARP1 (A) The ratio of Renilla luciferase to Firefly luciferase activity for HEK293T cells expressing TOP and mutant-TOP reporters bearing PEST motif in presence of Nsp1 or Nsp2 and Torin 1 or DMSO. The effect of Torin 1 was more pronounced for reporters that carry a PEST motif, which reduces the half life of the protein to ~20 min thereby reducing the pre-existing signal caused by its accumulation prior to Torin 1 treatment **(B)** The ratio of Renilla luciferase to Firefly luciferase activity in control HEK293T-Cas9 cells and LARP1 KO cells expressing EEF2 TOP

reporters with the PEST motif in the presence of Torin or DMSO. **(C)** The ratio of Renilla luciferase to Firefly luciferase activity in control; HEK293T-Cas9 cells and LARP1 KO HEK293T cells expressing minimal vector in presence of Nsp1 or Nsp2.

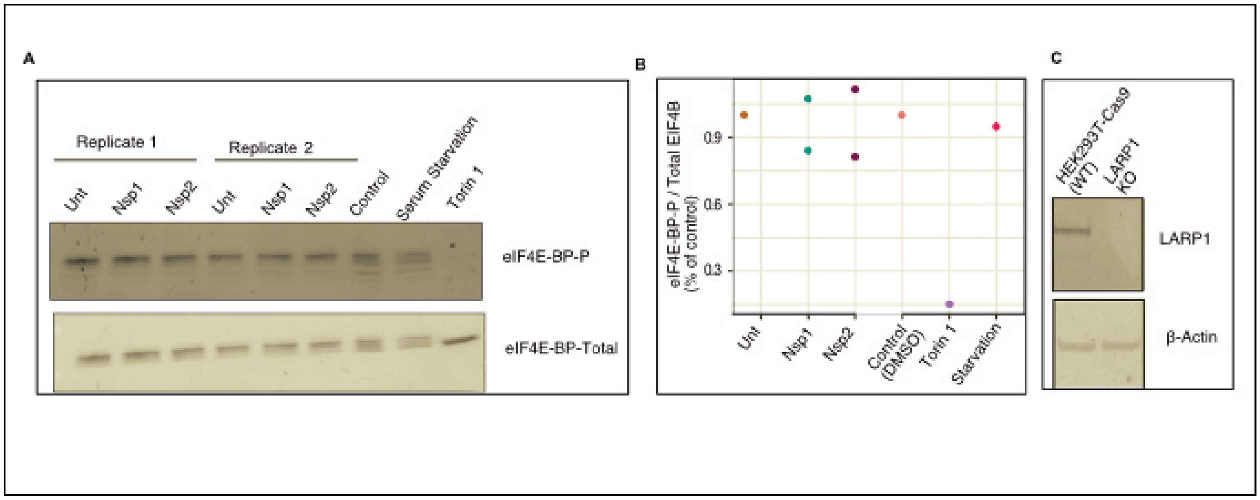

Supplemental Figure 25- mTOR activity remains unaffected upon expression of Nsp1 in HEK293T cells.

HEK293T cells were either Untransfected (Unt), or transfected with Nsp1 or Nsp2 followed by analysis of protein levels by Western blot. **(A)** depicts a representative western blot image for eIF4E-BP (pT37/46) and eIF4E-BP- total. Torin 1 and serum starved cells were used as controls **(B)** The ratio of eIF4E-BP (pT37/46) and eIF4E-BP- total normalised to that of untransfected cells are shown for individual experiments **(C)** shows the representative western band for LARP1 and β- actin. LARP1 KO HEK293T control cells and its corresponding control HEK293T-Cas9 was used as control for LARP1 protein detection
